# Aflatoxin M_1_ Determination in Infant Formulae Distributed in Monterrey, Mexico

**DOI:** 10.3390/toxins12020100

**Published:** 2020-02-04

**Authors:** Patricia A. Quevedo-Garza, Genaro G. Amador-Espejo, Rogelio Salas-García, Esteban G. Ramos-Peña, Antonio-José Trujillo

**Affiliations:** 1 Facultad de Salud Pública y Nutrición, Universidad Autónoma de Nuevo León, Monterrey 64460, N.L.; Mexico; patricia.quevedog@uanl.mx (P.A.Q.-G.); rogelio.salasg@uanl.mx (R.S.-G.); esteban.ramosp@uanl.mx (E.G.R.-P.); 2 CONACYT–Centro de Investigación en Biotecnología Aplicada-IPN, Ex-Hacienda San Juan Molino Carretera Estatal Tecuexcomac, Tlaxcala 90700, Mexico; genaroamador2014@gmail.com; 3 Centre d’Innovació, Recerca i Transferència en Tecnologia dels Aliments (CIRTTA), TECNIO-UAB, MALTA-Consolider Team, Departament de Ciència Animal i dels Aliments, Facultat de Veterinària, Universitat Autònoma de Barcelona, 08193 Bellaterra, Spain

**Keywords:** AMF_1_, infant formulae, estimated daily intake, carcinogenic risk index, Monterrey (Mexico)

## Abstract

The occurrence of aflatoxin M_1_ (AFM_1_) in infant formulae commercialized in the metropolitan area of Monterrey (Nuevo León, Mexico) was determined by using immunoaffinity column clean-up followed by HPLC determination with fluorimetric detection. For this, 55 infant formula powders were classified in two groups, starter (49 samples) and follow-on (6 samples) formulae. Eleven of the evaluated samples (20%) presented values above the permissible limit set by the European Union for infant formulae (25 ng/L), ranging from 40 to 450 ng/L. The estimated daily intake (EDI) for AFM_1_ was determined employing the average body weight (bw) of the groups of age in the ranges of 0–6 and 6–12 months, and 1–2 years. The results evidenced high intake values, ranging from 1.56 to 14 ng/kg bw/day, depending on the group. Finally, with the EDI value, the carcinogenic risk index was determined, presenting a high risk for all the evaluated groups. Based on these results, it is a necessary extra effort by the regulatory agencies to reduce the AFM_1_ presence in infant formulae consumed in Mexico.

## 1. Introduction

Aflatoxins’ presence in food products is one of the major health concerns of the regulatory agencies around the world. These toxins include around 20 metabolites produced by molds such as *Aspergillus flavus* and *A. parasiticus*, which is the most important of the aflatoxin B_1_ (AFB_1_) and is normally found in foods, especially those having high carbohydrate and/or fat contents [1]. Its occurrence has been reported in numerous food and feedstuff, including cereals and cereal-derived products [2].

Cattle feed with contaminated crops of AFB_1_ may lead to the formation of a hydroxylated metabolite named aflatoxin M_1_ (AFM_1_), which is excreted in the milk of lactating animals and whose name is due to the source detected [3]. Numerous researchers have reported a linear relationship of about 0.3–6.2% between the amount of AFM_1_ detected in milk and AFB_1_ in feed consumed by the animals [4]. Nevertheless, the percent of AFM_1_ excreted depends on various factors, including concentration of AFB_1_ in feed, milk yield, stage of lactation and breed [5].

Even though AFM_1_, the main monohydroxylated derivate of AFB_1_, presents less carcinogenic and mutagenic activity than AFB_1_, it exhibits a high level of genotoxic activity and certainly represents a health risk because of its elevated possibility of accumulation and binding to DNA [6]. Based on this, different health agencies such as the World Health Organization and the International Agency for Research on Cancer (IARC) have published articles in which AFM_1_ is a strong genotoxic and hepatotoxic agent [7]. Therefore, AFM_1_ has been evaluated as a possible human carcinogen agent, and although until 2002 it was classified in the 2B Group, with a tolerable daily intake (TDI) of 2 ng/kg bw [8], based on numerous scientific evidence that demonstrated carcinogenic and other (teratogenic, genotoxic and immunosuppressive) effects, it was reclassified into the first group [7].

Hence, the elimination of risk sources represents a major assignment for government agencies and food processors, not only for the contaminated products directly consumed by humans but also in feeding cattle that consume contaminated crops, whose products can reach the human being. Government regulations around the world concerning AFM_1_ limits differ from one other. The lowest AFM_1_ concentration was approved by the European Union (EU) and the Codex Alimentarius, fixing a maximum admissible level of 50 ng/L in fluid milk and dried or processed milk products [9,10]. On the contrary, higher AFM_1_ concentrations (500 ng/L) are permitted in the United States of America (USA) and some Latin American countries (such as Mexico and the MERCOSUR agreement), and China allows a maximum limit of 62.5 ng/L [11,12,13,14]. However, because of the higher susceptibility of infants to AFM_1_, the EU and the Codex Alimentarius fixed the maximum admissible level of 25 ng/L for infant formulae, follow-on formulae and dietary foods for medical purposes intended specifically for infants [9].

Another major problem concerning the presence of AFM_1_ in milk is the different dairy products it’s included in (e.g., liquid milk, yogurt, cheese, milk powder, ice cream, regular cream, among others) and the fact that the aflatoxin cannot be eliminated by regular heat treatments such as pasteurization or ultra-high temperature processing [15]. Besides, one of the most important products manufactured from milk are the infant formulae, in which there is significant risk of AFM_1_ intoxication because small amounts of this toxin in the product may represent an important portion of aflatoxin intake [16].

Despite the danger associated to the AFM_1_ presence in milk, only a few articles are available regarding the presence of this toxin in milk and dairy products in Mexico [17,18], and no studies have been published regarding its presence in infant formulae or intake assessment for AFM_1_ in the country. Based on this, the aim of this study was to evaluate the AFM_1_ occurrence in infant formulae and to estimate the exposure of infant milk consumers to AFM_1_ by means of a sampling of the infant formulae brands distributed in Monterrey (Nuevo León, Mexico).

## 2. Results and Discussion

### 2.1. Occurrence of AFM_1_ in Infant Formulae

Table 1 shows the results obtained from the analyzed samples, with 20% of them being positive for the toxin in a range of 40 to 450 ng/L, and an average AFM_1_ concentration of 40 ± 99 ng/L for all analyzed samples, which is higher than the limit established for AFM_1_ in infant formulae by the Codex Alimentarius (25 ng/L) [11]. Nevertheless, when the infant formulae were evaluated separately (starter and follow-on groups), it can be observed that the AFM_1_ values increased from one group to another. In the starter formulae, the percentage of samples exceeding the AFM_1_ limit was 14%, remarkably lower than the percentage of samples above the limit in the follow-on formulae (67%). Furthermore, the media in the starter formulae (20 ± 67 ng/L) was below the EU or Codex Alimentarius AFM_1_ limit (25 ng/L), compared to the follow-on formulae, with an average (180 ± 185 ng/L) exceeding the AFM_1_ limit. Although the AFM_1_ levels in starter formulae were significantly (*p* < 0.05) lower than those in follow-on formulae, it is important to notice the small number of samples evaluated in the follow-on formulae, compared to the infant formula evaluated in the starter group.

Regarding legislation about AFM_1_ limits in infant formulae, most of the countries do not have an established limit, which is the case of most of the Latin-American countries (including Mexico), which tends to apply the limit established by the Codex Alimentarius or the EU regulation (25 ng/mL) [11,19].

The occurrence of AFM_1_ in infant formulae varies in different countries. Gomez-Arranz and Navarro-Blasco [20] evaluated the presence of AFM_1_ in infant formulae in Spain, testing 69 samples and detecting the presence of AFM_1_ in 26% of them. In this case, all the detected samples were below the EU established limit. More recently, Akhtar et al. [21] determined the AFM_1_ presence in infant formulae in Pakistan, evaluating 13 samples, in which 53.84% of the samples were positive to the toxin presence and 30.76% exceeded the EU limit. Kanungo and Bhand [22] evaluated the AFM_1_ presence in infant formulae in India, determining that in 72 evaluated samples, all of them were above the EU permitted limit (25 ng/kg) and 75% of the samples exceeded the USA and Indian Food regulation limit (500 ng/kg). Er et al. [4] published a study evaluating the AFM_1_ presence in infant formula in Turkey, evaluating 84 samples with only one sample positive for the toxin. In this sense, Li et al. [14] detected the presence of AFM_1_ in powder base for infant formulae in China, evaluating a total of 1207 samples, with 56 samples being positive for the toxin without passing the Chinese limit (62.5 ng/kg). Awaisheh et al. [23] determined the AFM_1_ content in infant formulae (120 samples; 48 starter and 72 follow-on formulae) distributed in Jordan, with 58 positive samples for the toxin presence, with a media of 69 and 84 ng/kg for the starter and follow-on formulae, respectively.

### 2.2. Infant Formulae Daily Intake by Age Group

The present study is the first evaluation of the daily intake by Mexican minors, based on average consumption and body weight (Table 2). The Mexican Standard NOM-031-SSA2-1999 [24] classifies infants in two groups of infant formulae consumption: i) minor lactating (0–12 months), and ii) major lactating (one to two years). The consumption in these groups is starter and follow-on formulae for the first and the second year, respectively.

Based on the occurrence of AFM_1_ in infant formulae and the body weight of infants, the estimated daily intake (EDI) for AFM_1_ was in a range of 1.56 to 14 ng/kg bw per day, which represents the values estimated for one year-old infants when they are fed with starter or with follow-on formulae, respectively. However, when major lactating groups gain weight and reduce the follow-on formula intake (i.e., two years old), the EDI is reduced up to 4.28 ng/kg bw/day. Awaisheh et al. [23] have evaluated the infant formulae consumed in Jordan, presenting an EDI of 1.57 and 1.55 ng/kg bw/day for infants aged six and 12 months, respectively. On the other hand, Ismail et al. [25] reported an EDI value of 4.1 ng/kg bw/day for children aged one to three years in Pakistan. It is considerable the work developed by the food agencies seeking to reduce the presence of AFM_1_ in milk and infant formulae. In this sense, Oliveira et al. [26] published an article evaluating the presence of AFM_1_ in infant formulae in Brazil with a daily intake of 22 ng/kg bw/day. In contrast, almost 20 years later, Ishikawa et al. [27] determined the AFM_1_ presence in infant formulae in the same country, presenting an important reduction in EDI values (0.078–0.306 ng/kg bw/day). Likewise, lower EDI values than the present study were detected in infant formulae consumed in Spain (n = 69) (0.02–0.13 ng/kg bw/day) [4]. Further, Ruangwises et al. [28] evaluated AFM_1_ presence in milk powder distributed in Thailand (90 samples) showing EDI values of 0.16 ng/kg bw/day in milk consumed by infants up to three years.

Comparing the results of AFM_1_ occurrence in infant formulae and in breast milk in Mexico, the results are quite similar. Thereby, Cantú-Cornelio et al. [29] evaluated the presence of AFM_1_ in breast milk of nursing mothers in central Mexico (112 samples), with an EDI value of 2.35 ng/kg bw/day, comparable results to the values obtained in the present study. These results show the importance of evaluating the presence of AFB_1_ in different products consumed by nursing mothers in order to reduce the toxin that may be transformed into AFM_1_ and reach infants by breast milk.

Table 2 also presents the result of the carcinogenic risk index (CRI) for the evaluated population. At this day, up to our knowledge, no CRI study evaluating the infant population of Mexico has been published. The AFM_1_ ingestion obtained in this study was greater than the TDI value (2 ng/kg bw/day) calculated by Kuiper-Goodman [8] dividing the TD50 by the safety factor 5000, indicating that there is a potential high risk for liver cancer due to the consumption of infant formulae in Mexican consumers groups studied.

## 3. Conclusions

The results of the current study have shown a high presence of AFM_1_ in infant formulae distributed in the Monterrey (Mexico) metropolitan area. From fifty-five samples evaluated, 20% exhibited a toxin content above the EU and Codex Alimentarius limit (25 ng/L), presenting a range of 40–450 ng/L. Further, in classifying the samples by the type of infant formulae and infant age for consumption (starter formula for minor infants up to one year, and follow-on formula for major infants between one and two years), different levels of AFM_1_ were obtained (20 ng/L for starter and 180 ng/L for follow-on formulae). Besides, based on the average body weight of the evaluated groups, the EDI value was calculated, with values in the range of 1.56–14 ng/kg bw/day. Finally, with the EDI data, the CRI was determined, obtaining a result of risk in all the evaluated groups. Based on these results, an important effort should be carried out by the regulatory agencies and milk producers in order to reduce AFM_1_ levels in milk in general, and, in particular, in batches that will be employed for infant formulae elaboration because of the high cancer risk associated with AFM_1_ presence and the infant consumers’ vulnerability.

## 4. Materials and Methods

### 4.1. Sample Collection

Fifty-five infant formula samples from drug stores and supermarkets sold in Monterrey (Nuevo León, Mexico) were obtained. From these, 49 were starter formulae (0–12 months) and 6 were follow-on formulae (1–2 years). Among the starter formulae, 6 were pre-term formulae (formulated for prematurely born, regurgitation episodes by immature esophageal sphincter, or low birth weight infants), 11 were hypoallergenic formulae (specialized formula based on casein, whey or soy protein hydrolysates) and 9 were lactose free formulae (designed for lactose intolerant infants based on lactose hydrolysis by β-galactosidase or formulated from soy protein isolates).

All formulae were supplied as powder milks. Infant formula containers (cans or bags) were stored in dark at room temperature until analyses were performed. 

### 4.2. Sample Preparation

Powder-based formula samples were suspended in deionized warm water according to the manufacturer instructions. The method used for sample preparation and AFM_1_ determination was that specified by the method ISO 14,501 [30]. Suspended infant formula samples were centrifuged at 4200 × *g* for 15 min to separate and remove the milk fat. Aliquots of skimmed milk (50 mL) were filtered (Whatman no. 4 filter paper) and slowly passed (1–2 drops/s) through an immunoaffinity column (AflaM1 HPLC, VICAM, Milford, MA, USA) fitted on a vacuum manifold, and washed twice with 10 mL of distilled water. Thereafter, the AFM_1_ was eluted with 4 mL of acetonitrile, allowing a time contact of at least 60 s. The eluate was collected in amber vials, the solvent was evaporated in a water bath at 40 °C with nitrogen, and the residue reconstituted in water:acetonitrile (67:33) and filtered by Millipore filters (0.45 μm) in amber vials.

### 4.3. HPLC Analysis

The HPLC analysis was carried out in a Varian HPLC model 9012 (Agilent Technologies, Santa Clara, CA, USA) connected with a fluorescence detector Varian ProStar (Agilent Technologies Santa, Clara, CA, USA). The separation column was a Phenomenex C18 with 4.5 × 250 mm and 5 μm of particle size (Phenomenex, Torrance, CA, USA). Water and acetonitrile mixture were used as a mobile phase in a proportion of 67:33 (v/v), at a flow rate of 1 mL/min, and an injection volume of 100 μL. Fluorometric detection was achieved at 360 nm excitation and 440 nm emission wavelength.

To assess the performance of the analytical method, linearity, limits of detection (LOD) and quantification (LOQ), recovery and precision (repeatability) were studied. Linearity was evaluated using standard calibration curves that were constructed by plotting the peak area versus the analyte concentration. The calibration curves were established using eight levels of concentrations from LOQ to 100 times LOQ. The regression curve obtained was y = 287.78 x + 75.10 giving appropriate value for the linearity (R^2^ = 0.998). LOD (2 ng/L) and LOQ (5 ng/L) were calculated as the sample blank value plus 3 and 10 times its standard deviation, respectively. In order to determine the recovery, reconstituted milk was added with 3 levels of AFM_1_ concentrations (50, 100 and 200 ng/L). The obtained values of recovery were between 83% and 104%. The precision (15.18%) was calculated as repeatability by means of triplicates in each of the levels analyzed in the recovery assay.

### 4.4. Determination of AFM_1_ Exposure in the Population 

The determination of the exposure level or estimated daily intake (EDI) in the population of Monterrey to the AFM_1_ due to the consumption of infant formulae was carried out by combining data on the average daily consumption of milk by groups of age, with the average concentration of AFM_1_ found in this work, as well as the average body weight (bw) of the population by age groups. For this, Equation (1) was applied:

(1) $$\mathrm{Estimated}\;\mathrm{AFM}1\;\mathrm{daily}\;\mathrm{intake}\;\left(\frac{\mathrm{ng}}{\mathrm{kg}\;\mathrm{bw}}/\mathrm{day}\right)=\frac{\mathrm{Milk}\;\mathrm{intake}\;\left(L\right)\times \mathrm{AFM}1\left(\frac{\mathrm{ng}}{L}\right)}{\mathrm{Body}\;\mathrm{weight}\;\left(\mathrm{kg}\right)}$$

where: Milk intake is the average amount of milk that the infant population ingests daily, expressed in liters. AFM_1_ is the average concentration of AFM_1_ contained in the analyzed samples, expressed in ng/L. Body weight is the bw average in the population by age groups in kilograms.

The data corresponding to the daily milk consumption by age groups was obtained from the National Survey of Health and Nutrition of Mexico (ENSANUT) [31], in the section corresponding to Nuevo León State.

In order to obtain the daily intake of AFM_1_ in the infant population, it was necessary to separate the population, as indicated by Mexican Standard NOM-031-SSA2-1999 [24] in: (1) minor lactating (newborn up to 6 months), at this stage of the infant’s life, their diet is only based on breast milk or infant formulae for initiation; (2) minor lactating (from 6 to 12 months), at this stage, ablactation occurs, and the starter infant formulae and dairy infant formulae containing cereals and honey are taken as the infant diet at this stage of life; (3) major lactating (from 12 to 24 months), at this stage the dairy intake is determined by the follow-on formulae and those containing cereals and honey. From the ENSANUT [31] survey, the average weights of the infant population (minor and major lactating) were obtained.

Likewise, the CRI was estimated based on the proposal of Kuiper-Goodman [8], which estimates the TDI of AFM_1_ by dividing the TD50 (threshold dose by body weight; 10,380 ng/kg bw per day for AFM_1_) by the safety factor 5000, to give an estimated value of 2 ng/kg bw per day. A CRI of AFM_1_ higher than 2 ng/kg bw indicates liver cancer risk to consumers [8,32].

### 4.5. Statistical Analysis

All infant formulae were analyzed in duplicates. Collected data were statistically evaluated using the nonparametric Wilcoxon rank sum test with continuity correction of R Core Team (Vienna, Austria) [33]. AFM_1_ concentrations were expressed as mean ± standard deviation in order to show the occurrence of the toxin in infant formulae.

## Figures and Tables

**Table 1:** Aflatoxin M_1_ presence in infant formulae.

Infant Formulae	*N*	Positive Samples	AFM_1_ (ng/L)	
			Range	Mean ± SD
Total of samples	55	11 (20%) *	0.00–450	40 ± 99
Starter formula	49	7 (14%)	0.00–420	20 ± 67 ^b^
Follow-on formula	6	4 (67%)	0.00–450	180 ± 185 ^a^

* Value in parentheses indicates the samples percentage above the limit set by the Codex Alimentarius (25 ng/L) with respect to the total. ^a,b^ Different online letters indicate significant mean differences among the different types of infant formulae (*p* < 0.05).

**Table 2:** Estimated aflatoxin M_1_ daily intake by average body weight and carcinogenic risk index (CRI) in children population based on the ENSANUT (2012).

Age (years)	Average Body Weight (bw) (kg)	Intake Type	Average Consumption		* CRI (2 ng/kg bw/day)
			Infant Formula Intake Range (L/day)	AFM_1_ Intake (ng/kg bw/day)	
0–0.5	3.55–7.3	Starter infant formula	0.78–0.93	4.39–2.55	Risk
0.5–1	7.3–10.8	Starter infant formula	0.93–0.84	2.55–1.56	Risk
1–2	10.8–13.03	Follow-on infant formula	0.84–0.31	14–4.28	Risk

* According to the Kuiper-Goodman equation [8].

## Abbreviations

AFB_1_	Aflatoxin B_1_
AFM_1_	Aflatoxin M_1_
bw	Body weight
CRI	Carcinogenic risk index
EDI	Estimated daily intake
EU	European Union
HPLC	High Performance Liquid Chromatography
TDI	Tolerable daily intake
USA	United States of America
